# Prognostic Impact of Circulating Methylated Homeobox A9 DNA in Patients Undergoing Treatment for Recurrent Ovarian Cancer

**DOI:** 10.3390/cancers14071766

**Published:** 2022-03-30

**Authors:** Louise Faaborg, Rikke Fredslund Andersen, Marianne Waldstrøm, Jon Røikjær Henriksen, Parvin Adimi, Anders Jakobsen, Karina Dahl Steffensen

**Affiliations:** 1 Department of Oncology, Lillebaelt Hospital, University Hospital of Southern Denmark, Beriderbakken 4, 7100 Vejle, Denmark; parvin.adimi@rsyd.dk (P.A.); anders.jakobsen@rsyd.dk (A.J.); karina.dahl.steffensen@rsyd.dk (K.D.S.); 2 Department of Regional Health Research, University of Southern Denmark, J.B. Winsløws Vej 19, 5000 Odense C, Denmark; 3 Department of Clinical Biochemistry and Immunology, Lillebaelt Hospital, University Hospital of Southern Denmark, Beriderbakken 4, 7100 Vejle, Denmark; rikke.fredslund.andersen@rsyd.dk; 4 Department of Pathology, Lillebaelt Hospital, University Hospital of Southern Denmark, Beriderbakken 4, 7100 Vejle, Denmark; marianne.waldstroem@rsyd.dk; 5 Department of Oncology, Odense University Hospital, J.B. Winsløws Vej 4, 5000 Odense C, Denmark; jon.henriksen@rsyd.dk

**Keywords:** ovarian cancer, liquid biopsy, circulating tumor DNA, Homeobox A9, methylation, biomarker, prognosis, relapse

## Abstract

**Simple Summary:**

Ovarian cancer remains a clinical challenge with considerable mortality. Circulating tumor DNA (ctDNA) has been suggested as a prognostic biomarker and enables the longitudinal evaluation of a patient’s disease and response to treatment. However, the role of ctDNA in treatment monitoring and for guiding treatment decisions in ovarian cancer remains unclear. We aimed to examine a gene methylation biomarker in the plasma of patients suffering a relapse of ovarian cancer in order to investigate prognostic potential and identify patients most likely to benefit from treatment, measured by overall survival. In the study, the methylated gene *HOXA9* was found to be significantly related to poor survival, with the potential to observe the progression of the disease at an early stage and spare patients from ineffective treatment. Monitoring ctDNA during treatment is clinically feasible, further efforts are, however, required for standardization and for demonstrating improvement in treatment management.

**Abstract:**

Methylated *Homeobox A9* circulating tumor DNA (meth-*HOXA9*) has been suggested as a blood-based biomarker in epithelial ovarian cancer (EOC), although its prognostic significance remains unproven. The aim of the present study was to investigate the prognostic impact of meth-*HOXA9* in patients with recurrent EOC. DNA was purified from 4 mL plasma and, following bilsulfite conversion, meth-*HOXA9* was analyzed using a methylation-specific droplet digital PCR. Detection of meth-*HOXA9* was reported as a percentage of total DNA and as a binary variable (detectable and undetectable). Meth-*HOXA9* status and its dynamics during palliative treatment were correlated with overall survival (OS) as the primary endpoint. At baseline, meth-*HOXA9* was detected in 65.9% (83/126) of the patients. The median OS was 8.9 and 17.9 months in patients with detectable and undetectable meth-*HOXA9* at baseline (hazard ratio: 2.04, *p* = 0.002), which remained significant in the multivariate analysis. Median OS in patients with an increase in meth-*HOXA9* after one treatment cycle was 5.3 months compared to 33 months in patients with undetectable meth-*HOXA9* (*p* < 0.001). Meth-*HOXA9* was significantly related to poor survival and may serve as a prognostic marker in patients with recurrent EOC. The longitudinal monitoring of meth-*HOXA9* is clinically feasible with the perspective of aiding clinical decision making.

## 1. Introduction

Recurrent epithelial ovarian cancer (EOC) remains a clinical challenge with considerable mortality. In contrast to first-line chemotherapy, treatment of recurrent EOC is less standardized [1,2], and there are numerous chemotherapeutic agents to offer these patients. Although response rates to treatment are low (10–25%) [3], and short progression-free survival is a characteristic of recurrent EOC, most patients have a wish for further palliative treatment. Hence, the early prediction of response or resistance to chemotherapy could have important implications for clinical management and quality of life.

Patients differ concerning response to chemotherapy, progression-free survival, and overall survival; identifying the subset of patients who will benefit from treatment would be a step towards an individualized treatment strategy. An applicable biomarker to support such treatment decisions is lacking. Currently, response to treatment and the pertaining clinical decisions are based on the Response Evaluation Criteria in Solid Tumours (RECIST) and cancer antigen 125 (CA125), but these measures are inadequate. Inter-reader variability and peritoneal carcinomatosis make evaluation by imaging difficult, and since imaging is performed after several treatment cycles, months of ineffective treatment may be the result. The lack of expression of CA125 in a proportion of patients [4,5], combined with the challenges of monitoring platinum-resistant EOC [6,7], makes CA125 an inappropriate evaluation marker.

Although *BRCA 1/2* and *TP53* mutations are frequent in EOC, one consistent mutation is lacking, mainly due to the molecular heterogeneity and low mutational load in EOC [8,9,10]. Moreover, tumor subclones may arise during disease progression and alter the pattern and proportion of aberrations between the primary tumor and metastases [11]. Analysis of circulating tumor-specific DNA (ctDNA) bypasses these issues because it is anticipated to be released from multiple tumor regions, and reflects both intratumoral heterogeneity and evolution, with the perspective of repeated measurement for the evaluation of disease progression and treatment response. The use of assays assessing genomic variants of ctDNA in EOC is increasing [12,13], but the potential of ctDNA assays for monitoring treatment response in patients with EOC has only been sparsely examined [14].

Aberrant methylation patterns are detectable in the majority of malignant tumor cells and methylated ctDNA has, to some extent, been examined in EOC. For individual genes, however, the diversity of methylation profiles and frequency of methylation detection vary greatly between studies [15], and the reported changes remain unverified by independent studies. Methylation of the Homeobox A9 gene (*HOXA9*) has especially been associated with EOC [16,17,18], but its role in treatment decisions and monitoring is unexplored.

The HOXA genes coordinate the patterns of the Müllerian system during embryogenesis, with *HOXA9* normally expressed in the fallopian tubes [19] and was selected as the methylation marker of interest based on the previous findings [16,17,18] and small studies from our group [20,21].

In this study, we monitored methylated *HOXA9* ctDNA (meth-*HOXA9*) in patients with recurrent EOC during chemotherapy. We included all histopathological subtypes despite heterogeneity of disease, as we wanted to investigate meth-*HOXA9* ctDNA as a general, consistent marker of EOC. The aim was to identify patients who will benefit from chemotherapy as measured by overall survival and progression-free survival.

We found that meth-*HOXA9* could be used as a universal prognostic biomarker in recurrent EOC, with the potential to stop ineffective treatment earlier—an increase in meth-*HOXA9* after one treatment cycle is highly prognostic of outcome. The results of this study could represent a useful tool to support clinical decision making in recurrent EOC patients.

## 2. Materials and Methods

The reporting of this study follows the REMARK guidelines (REporting recommendations for tumor MARKer prognostic studies) [22] as recommended by the National Cancer Institute [23].

The study was approved by the Regional Committee on Health Research Ethics for Southern Denmark (S-20160049) and the Danish Data Protection Agency (16/28860). All participating patients provided written informed consent at inclusion.

### 2.1. Patient Eligibility

The study cohort consisted of 126 consecutively enrolled patients with recurrent EOC, who were offered palliative chemotherapy according to institutional and national guidelines at the Department of Oncology, Lillebaelt Hospital, Vejle, Denmark, between December 2016 and April 2021. Blood samples were prospectively collected for analysis of overall survival as the primary endpoint and progression-free survival and treatment response as the secondary endpoints.

The main inclusion criteria were recurrence of histologically verified epithelial EOC, evaluable disease by RECIST [24] and/or by the Gynecological Cancer Intergroup (GCIG) CA125 criteria [25], age >18 years, performance status ≤2, and life expectancy >3 months.

### 2.2. Analysis of Meth-HOXA9

Blood samples were collected before treatment initiation and at every treatment cycle until progression or the stopping of treatment for other reasons.

Details on DNA isolation and meth-*HOXA9* analysis have been described previously [26,27] and are available in the Supplementary Materials [28,29], with details on primers and probes described in Table S1. In short, ctDNA was extracted from 4 mL plasma, and bisulfite converted following droplet digital PCR (ddPCR) analysis using an in-house designed methylation-specific assay (*HOXA9*) and a control assay (Albumin) described in the reference [29].

Plasma from 100 self-reported healthy donors was used to establish the limit of blank and cut-off for a positive sample (meth-*HOXA9* detectable). Results were reported as number of droplets containing meth-*HOXA9* accepting a <5% false positive rate [30], which resulted in a cut-off of ≥5 meth-*HOXA9*-containing droplets equaling a positive test, whereas samples with lower values were considered undetectable. 

After determination of the cut-off for a positive sample, meth-*HOXA9* was reported as a percentage of total DNA ((meth-*HOXA9* copies/albumin copies) × 100) including a 95% confidence interval (CI) derived from the Poisson distribution and as detectable/undetectable (dichotomized). Meth-*HOXA9* was considered undetectable if the lower 95% CI included 0. 

The dynamics of meth-*HOXA9* during treatment were evaluated using the percentage of meth-*HOXA9* and considered stable if the 95% CI of the meth-*HOXA9* measurement was within the 95% CI of the previous measurement, decreasing if the measurement was below the 95% CI of the previous measurement but still detectable, and increasing if the 95% CI of the measurement was above the 95% CI of the previous measurement.

Analysis of meth-*HOXA9* was performed as blinded to the clinical endpoints.

### 2.3. Treatment Efficacy

Response to treatment was evaluated by Computed Tomography (CT) scans and CA125. At the time of enrollment, a CT scan was performed for the evaluation of disease according to the RECIST criteria. CT scans were repeated after every three treatment cycles (every 8–12 weeks). Patients receiving at least three treatment cycles were hence eligible for CT response evaluation unless progression had occurred before the planned assessment date. CA125 was analyzed at baseline and within five days before each treatment cycle with response defined according to the GCIG CA125 criteria. Treatment continued until progression, intolerable side effects, or the patient requesting discontinuation.

### 2.4. Statistical Analysis

Progression-free survival and overall survival was calculated from the start of each treatment cycle (first, second or third) to date of progression and/or death of any cause. Follow-up was censored at the time of data workup (July 2021). Kaplan-Meier curves illustrated survival, and log-rank statistics were used for comparison of survival plots. Multivariate survival analysis was performed using the Cox regression model with the proportional hazard assumption tested. The parameters entered in the multivariate Cox regression analysis were variables with a *p*-value < 0.1 in the univariate Cox regression analysis.

Categorical and continuous variables are presented as frequencies and means, respectively. Comparisons between groups were made with Wilcoxon rank-sum test for numeric non-parametric variables and Student’s *t*-test for numeric parametric data. Fischer’s exact test and chi-squared test were used for binary parametric data to compare two unpaired groups. Spearman correlation was used to consider correlations between meth-*HOXA9* and age and CA125 levels, respectively. All statistical analyses were performed using Stata/IC version 16^®^ (Stata-Corp LLC, College Station, TX, USA).

## 3. Results

### 3.1. Patient Characteristics

The majority of the 126 patients had high-grade serous carcinoma (*n* = 108, 85.7%). Half of the patients had received only one line of chemotherapy at enrollment, of which 25 patients (39.7%) had platinum-resistant disease and 38 patients (60.3%) had platinum-sensitive disease. The majority of patients were considered platinum resistant (*n* = 77, 61.1%). Baseline patient characteristics and the relation to meth-*HOXA9* status are outlined in Table 1. The mean time between initial diagnosis of EOC and first treatment in the present study was 40 months (median = 26.5 (range 2–210)) and the mean number of treatment cycles was 4.4 (median = 5 (range 1–24)). The treatment regimen stated in Table 1 was the primary treatment, as 25 patients (19.8%) received maintenance therapy with Bevacizumab (*n* = 16) or PARP inhibitors (*n* = 9) after the specified chemotherapy regimen.

### 3.2. Meth-HOXA9

At baseline, meth-*HOXA9* was detected in 65.9% (83/126) of the patients. A difference in age, CA125 and treatment regimen was found between patients with and without detectable meth-*HOXA9* (Table 1). Applying Spearman rank correlation coefficient, however, no correlation between meth-*HOXA9* and CA125 or age was found (rho = 0.279 and 0.196). After one treatment cycle, 64% (73/114) of the patients had detectable meth-*HOXA9*, which dropped to 60% (60/100) after three treatment cycles.

### 3.3. Prognostic Role of Meth-HOXA9 in Recurrent OC

The prognostic value of meth-*HOXA9* was analyzed at baseline (*n* = 126), at the second treatment cycle (*n* = 114) and after three cycles of treatment (*n* = 100). The 34 patients still alive at the time of analysis (July 2021) had a median follow-up of 16.3 months [range 2.4–47.2].

**Figure 1:**
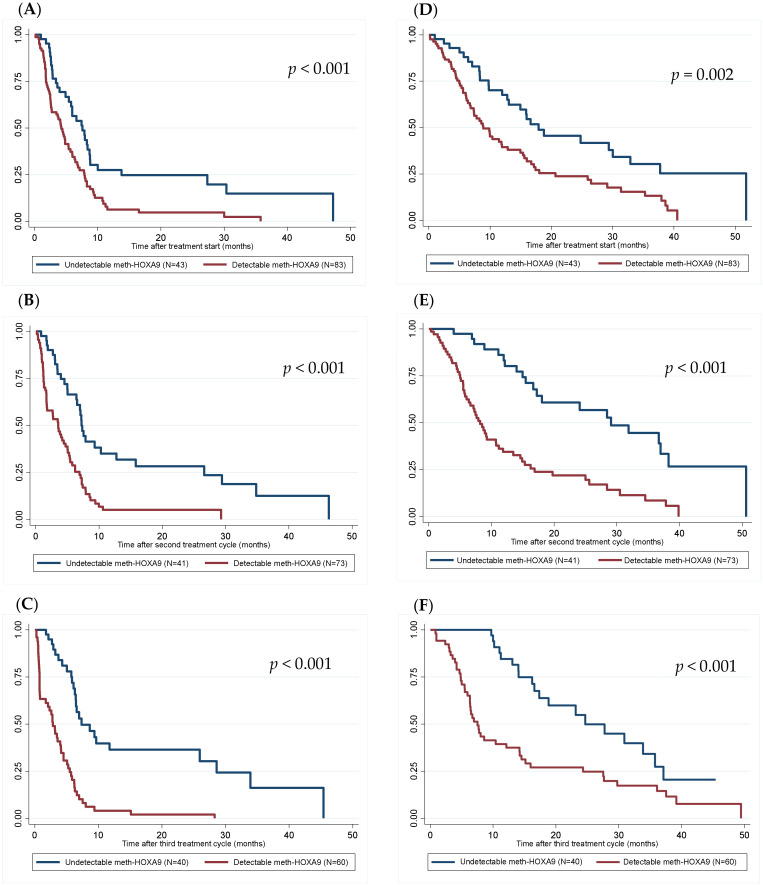
Kaplan-Meier plots for detectable vs. undetectable meth-*HOXA9* at baseline ((**A**)—PFS at baseline, (**D**)—OS at baseline), at second treatment cycle ((**B**)—PFS at second treatment cycle, (**E**)—OS at second treatment cycle), and at first evaluation after three treatment cycles ((**C**)—PFS after three treatment cycles, (**F**)—OS after three treatment cycles) for progression-free survival (**A**–**C**) and overall survival (**D**–**F**) (months).

The median overall survival in patients with detectable and undetectable meth-*HOXA9* at baseline was 8.9 and 17.9 months (logrank *p* = 0.002, Hazard ratio (HR) = 2.04, 95% CI: 1.29–3.23, Figure 1). At the second treatment cycle (3–4 weeks after baseline), the difference was even more pronounced (8.5 vs. 29 months, logrank *p* < 0.001, HR = 3.3, 95% CI: 1.95–5.5). This also applied to progression-free survival with a median of 4.2 and 7.6 months at baseline (*p* < 0.001) in patients with detectable and undetectable meth-*HOXA9*, respectively, and 3.5 and 7.2 months at the second treatment cycle (*p* < 0.001).

At the first evaluation after three treatment cycles, the difference in overall survival remained significant with a median overall survival of 7.5 and 24.7 months in patients with detectable and undetectable meth-*HOXA9*, respectively (logrank *p* < 0.001, HR = 2.54, 95% CI: 1.48–4.36).

In addition to meth-*HOXA9* status performance status, platinum sensitivity, previous lines of chemotherapy, and CA125 > 500 kUI/L were correlated with overall survival in the univariate analysis (Supplementary Materials, Table S2) and included in the multivariate analysis, in which meth-*HOXA9* remained prognostic of outcome at baseline (HR: 1.89, *p* = 0.008), at second treatment cycle (HR: 3.06, *p* < 0.001), and at status after three treatment cycles (HR: 2.17, *p* = 0.013, Table 2).

The univariate and multivariate Cox regression analyses were repeated in the subgroup of patients with high-grade serous carcinomas, in which meth-*HOXA9* remained significant of outcome (Supplementary Materials, Tables S3 and S4).

### 3.4. Meth-HOXA9 Dynamics during Treatment

Meth-*HOXA9* dynamics were evaluated after the first, second and third treatment cycle. Of the 114 patients evaluable after the first treatment cycle, 17 (14.9%) had an increase in meth-*HOXA9* (above the 95% CI of the baseline level), 38 (33.3%) had stable meth-*HOXA9*, 34 (29.8%) had undetectable meth-*HOXA9* of which the status changed from detectable to undetectable in 6, and 25 patients (21.9%) had a decrease in meth-*HOXA9* (below the 95% CI of the baseline level) (Table 3). Since only four and six patients had a decrease in meth-*HOXA9* after the second and third treatment cycle, this group was merged with the “stable” group in the survival analysis.

Patients with an increase in meth-*HOXA9* after one treatment cycle had a median overall survival of 5.3 months, compared to 11.9 months in patients with stable or decreasing meth-*HOXA9* and 33 months in patients with undetectable meth-*HOXA9* (*p* < 0.001). An increase after the second and third treatment cycle continued to be prognostic with an overall survival of 5.5 months, while undetectable meth-*HOXA9* remained favorable (overall survival = 29.1 and 24.7 months, respectively, *p* < 0.001 and *p* = 0.050).

**Figure 2:**
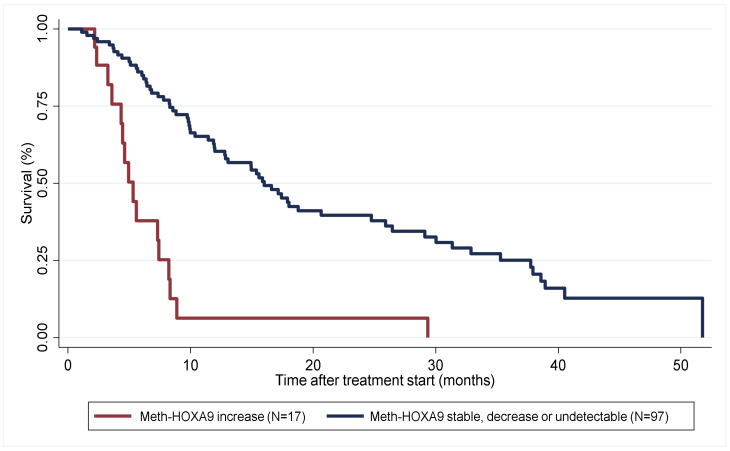
Kaplan-Meier plot illustrating overall survival for patients with an increase in meth-*HOXA9* (red, *n* = 17) vs. patients with stable, decreasing or undetectable meth-*HOXA9* (blue, *n* = 97) after one treatment cycle (months).

If patients with stable, decreasing and undetectable meth-*HOXA9* were grouped (*n* = 97) and compared to patients with an increase in meth-*HOXA9* (*n* = 17) after one treatment cycle, an overall survival of 16 and 5.3 months, respectively, was found (*p* < 0.001, Figure 2).

### 3.5. Meth-HOXA9 and Platinum-Resistant Disease

At baseline, meth-*HOXA9* was detected in 68.8% (53/77) of the patients with platinum-resistant disease. The median overall survival in platinum-resistant patients with detectable and undetectable meth-*HOXA9* at baseline was 6.7 and 12.8 months (*p* = 0.0274). At the second treatment cycle, the fraction of patients with detectable meth-*HOXA9* was 71.6% (48/67), in which the median overall survival was 6.4 months compared to 18 months in patients with undetectable meth-*HOXA9* at the second treatment cycle (*p* = 0.0012).

This also applied to progression-free survival with a median of 2.6 and 4.9 months at baseline (*p* = 0.007) in patients with detectable and undetectable meth-*HOXA9*, respectively. At the second treatment cycle, the difference in progression-free survival was even more pronounced in the subgroup of patients with platinum-resistant disease (1.7 vs. 6.4 months, logrank *p* < 0.001, HR = 3.06, 95% CI: 1.64–5.71).

Of 67 patients with platinum-resistant disease and evaluable meth-*HOXA9* dynamics after the first treatment cycle, 14 patients (20.9%) had an increase in meth-*HOXA9* compared to three patients (6.4%) in the subgroup of patients with platinum-sensitive disease.

An increase in meth-*HOXA9* after one treatment cycle in patients with platinum-resistant disease was significantly related to poor survival with a median overall survival of 5 months, compared to 8.6 months in patients with stable or decreasing meth-*HOXA9*, and 18.8 months in patients with undetectable meth-*HOXA9* (*p* < 0.001).

### 3.6. Treatment Efficacy

Of 97 patients with evaluable imaging after three treatment cycles, 37 (38.1%) had partial response, 39 (40.2%) had stable disease and 21 (21.6%) had progression. Twenty-four patients (19.0%) had progression before evaluable imaging after three treatment cycles, out of which nine cases were confirmed by imaging. Due to the withdrawal of consent and poor general condition, five patients were non-evaluable by imaging after three cycles. Of the 77 patients with platinum-resistant disease, 52 (67.5%) were evaluable after three cycles of treatment, of which 10 (19.2%) had partial response.

Forty-three patients (34.1%) were non-evaluable by CA125, i.e., CA125 < 70 kUI/L throughout the treatment course (*n* = 29), or the number of measurements was ≤2 (*n* = 14). Applying Cox regression overall survival was not related to response by CA125 (HR = 1.30, *p* = 0.150).

Baseline meth-*HOXA9* and its dynamics from baseline to the second treatment cycle are correlated to response by imaging and CA125 in Supplementary Materials, Table S5.

## 4. Discussion

This translational study was initiated to examine meth-*HOXA9* as a prognostic biomarker. Meth-*HOXA9* was found to be significantly correlated to clinical outcomes in patients undergoing treatment for recurrent EOC. Patients with detectable meth-*HOXA9* at baseline had significantly poorer overall survival after one and after three treatment cycles compared to those with undetectable meth-*HOXA9*. Furthermore, patients with an increase in meth-*HOXA9* after one treatment cycle had significantly reduced overall survival compared to patients having stable, decreasing, or undetectable meth-*HOXA9*. The findings applied both platinum-sensitive and platinum-resistant patients, though patients with platinum-resistant disease and detectable meth-*HOXA9* at baseline or at second treatment cycle had a remarkably short progression-free survival (2.6 and 1.7 months).

Mainly for diagnostic purposes, methylated *HOXA9* has previously been investigated in tissue [16,17] and in plasma/serum [18,31], and a recent study examined its presence in ascites [32] of patients with EOC. To our knowledge, no other studies than the small ones from our group [20,21] have explored the correlation of meth-*HOXA9* with prognosis.

A considerable number of studies, including ongoing clinical trials, examine ctDNA in relation to monitoring treatment response in EOC [12,33]; however, to date, no ctDNA-related test has been approved and evidence for using ctDNA to guide clinical decision making is weak [34]. In a study by Oikkonen et al., response to therapy using two or three consecutive ctDNA samples during treatment of EOC was examined, with the possibility of the rapid discovery of resistant cell populations and the early detection of recurrence [35]. The study applied a ctDNA workflow detecting >500 cancer-related genes, but included only 12 patients all with high-grade serous adenocarcinomas. This proof-of-principle study highlights, however, the potential to identify poor-responding patients after first cycles of chemotherapy using longitudinal ctDNA samples.

Recurrent EOC represents a heterogeneous group of patients with varying prognoses and unpredictable response to further treatment, for which the identification of poor-responders will be of great importance. EOC that relapse within 6 months of platinum treatment represents an extraordinary clinical challenge with low response rates to therapy and no predictive tests or signature to identify patients who will respond to specific drugs.

In the present study, an increase in meth-*HOXA9* after one cycle of treatment indicated a significantly reduced overall survival compared to undetectable meth-*HOXA9*, throughout histopathology and platinum status. The stable or decreasing parameter was not as favorable as the undetectable one. These findings emphasize that ctDNA dynamics can provide real-time therapeutic guidance, predict prognosis, and evaluate treatment resistance ahead of imaging, with the possibility to stop an inefficient treatment earlier for improved outcome and quality of life.

In the recurrent setting, a patient may be better served with the best supportive care rather than active anti-cancer treatment, which is justified only if there is a reasonable chance of benefit. Despite the heterogeneous group of patients in our study, meth-*HOXA9* could serve as a prognostic marker across types of treatment and platinum sensitivity with the potential to observe progression at an early stage and reduce the number of chemotherapy cycles without affecting survival.

As only a minor subgroup of patients with recurrent EOC, and especially platinum-resistance, will benefit from treatment [3], there is an obvious need for a new marker to select these patients. The fact that 34.1% of the patients (*n* = 43) in our study were non-evaluable by CA125, and since CT scanning has several limitations such as carcinomatosis, costs, inter-operator and/or inter-reader variability, underlines the need for a new biomarker to support treatment decisions.

Even though the plasma samples were prospectively collected with the purpose of biomarker analysis, the primary limitation of this study is its retrospective nature. The samples were stored for up to four years, which could have affected the amount of DNA [36]. In addition, the quantitative measurements of ctDNA represent a technical challenge, as the amount of circulating DNA might fluctuate over time and be susceptible to chemotherapy and albumin status. Quantification of meth-*HOXA9*, however, was performed in a single laboratory. This strength secures uniformity, reproducibility, and provides the proper validation of analytical variation using only one methylation marker applied to a large proportion of patients.

One of the major issues of incorporating ctDNA in the monitoring of treatment efficacy is that despite the evidence of clinical validity, there is no evidence of clinical utility [34]. Moreover, there is currently no consensus on how to evaluate and report ctDNA during treatment, and several different definitions have been reported, e.g., ‘relative change from baseline’, ‘x-fold reduction/increase’ and statistical calculations, with comparisons and validation of the definitions still lacking [21,37,38,39].

Further efforts are required for the standardization of the ctDNA analysis and for demonstrating improvement in treatment management with the use of the assay compared to not using it. To secure clinical validity, the data need to be validated in prospective, ideally randomized trials powered specifically for meth-*HOXA9* as a biomarker [23], and with patient management guided by meth-*HOXA9* analysis.

## 5. Conclusions

In conclusion, patients with detectable meth-*HOXA9* at treatment initiation had a significantly shorter progression-free survival and overall survival than those with undetectable meth-*HOXA9*. Outlining a high-risk population using meth-*HOXA9* prior to treatment initiation or after just one cycle of treatment is an attainable method of screening, with the potential to spare patients of ineffective treatment.

Although meth-*HOXA9* is in its infancy and awaiting validation in randomized controlled trials, it seems to be a marker of clinical response that could facilitate a personalized treatment approach.

## Figures and Tables

**Table 1:** Patient characteristics and status of meth-*HOXA9* at baseline.

Variable	All Patients (*n* = 126)	Detectable Meth-*HOXA9* (*n* = 83)	Undetectable Meth-*HOXA9* (*n* = 43)	*p*-Value
Age, mean (range)	68 (38–92)	69 (47–92)	65 (38–80)	0.021
FIGO stage at diagnosis				
I	4 (3.2%)	2 (2.4%)	2 (4.7%)	
II	4 (3.2%)	2 (2.4%)	2 (4.7%)	
III	41 (32.5%)	25 (30.1%)	16 (37.2%)	
IV	77 (61.1%)	54 (65.1%)	23 (53.5%)	0.524
Histology				
Low-grade serous	7 (5.6%)	4 (4.8%)	3 (7.0%)	
High-grade serous	108 (85.7%)	72 (86.7%)	36 (83.7)	
Endometrioid	4 (3.2%)	3 (3.6%)	1 (2.3%)	
Mucinous	3 (2.4%)	2 (2.4%)	1 (2.3%)	
Clear cell	2 (1.6%)	1 (1.2%)	1 (2.3%)	
Other	2 (1.6%)	1 (1.2%)	1 (2.3%)	0.851
CA125 (kUI/L), mean (range)	1220 (6–30,072)	1656 (6–30,072)	405 (11–3600)	0.003
Previous lines of chemotherapy				
1	63 (50.0%)	36 (43.4%)	27 (62.8%)	
2–3	49 (38.9%)	36 (43.4%)	13 (30.2%)	
4–5	14 (11.1%)	11 (13.3%)	3 (7.0%)	0.111
Platinum sensitive				
Yes	49 (38.9%)	30 (36.1%)	19 (44.2%)	
No	77 (61.1%)	53 (63.9%)	24 (55.8%)	0.38
Treatment regimen				
Carboplatin	21 (16.7%)	15 (18.1%)	6 (14.0%)	
Carboplatin + Liposomal Doxorubicin	27 (21.4%)	14 (16.9%)	13 (30.2%)	
Carboplatin + Paclitaxel	1 (0.79%)	1 (1.2%)	0 (0.0%)	
Liposomal Doxorubicin	25 (19.4%)	11 (13.3%)	14 (32.6%)	
Topotecan	29 (23.0%)	22 (26.5%)	7 (16.3%)	
Treosulfan	14 (11.1%)	13 (15.7%)	1 (2.3%)	
Paclitaxel (weekly)	4 (3.2%)	3 (3.6%)	1 (2.3%)	
Gemcitabine	3 (2.4%)	2 (2.4%)	1 (2.3%)	
Vinorelbine	1 (0.79%)	1 (1.2%)	0 (0.0%)	
Bevacizumab (monotherapy)	1 (0.79%)	1 (1.2%)	0 (0.0%)	0.04
Performance status				
0–1	98 (77.8%)	63 (75.9%)	35 (81.4%)	
2	28 (22.2%)	20 (24.1%)	8 (18.6%)	0.482
*BRCA 1/2* status				
*BRCA 1* positive	18 (14.3%)	13 (15.7%)	5 (11.6%)	
*BRCA 2* positive	5 (4.0%)	4 (4.8%)	1 (2.3%)	
*BRCA 1/2* negative	75 (59.5%)	49 (59.0%)	26 (60.5%)	
Unknown *BRCA* status	28 (22.2%)	17 (20.5%)	11 (25.6%)	0.812
BMI, mean (range)	25 (16–44)	25 (16–44)	26 (20–42)	0.125

**Table 2:** Multivariate Cox regression analyses.

Variable	OS, Baseline (*n* = 126)		OS, Second Treatment Cycle 2 (*n* = 114)		OS After Three Treatment Cycles (at Response Evaluation, *n* = 100)	
	HR (95% CI)	*p*-Value	HR (95% CI)	*p*-Value	HR (95% CI)	*p*-Value
Meth-*HOXA9* status						
Undetectable	Reference		Reference		Reference	
Detectable	1.89 (1.18–3.01)	0.008	2.99 (1.73–5.18)	<0.001	2.17 (1.18–3.98)	0.013
Performance status						
0–1	Reference		Reference		Reference	
2	3.16 (1.93–5.20)	<0.001	2.56 (1.41–4.65)	0.002	2.73 (1.36–5.48)	0.005
Platinum sensitive						
No	Reference		Reference		Reference	
Yes	0.44 (0.26–0.73)	0.002	0.51 (0.29–0.88)	0.015	0.72 (0.40–1.29)	0.270
Previous lines of chemotherapy						
1–3	Reference		Reference		Reference	
4–5	2.68 (1.40–5.15)	0.003	3.30 (1.56–6.98)	0.002	3.52 (1.47–8.41)	0.005
CA125 (kUI/L), at baseline						
>500 kUI/L	Reference		Reference		Reference	
≤500 kUI/L	0.74 (0.47–1.16)	0.185	0.92 (0.56–1.52)	0.747	0.82 (0.47–1.42)	0.477

OS, overall survival; HR, hazard ratio; CI, confidence interval.

**Table 3:** Dynamics of meth-*HOXA9* during treatment and the correlation with overall survival.

Time of Evaluation	Meth-HOXA9 Increase	Meth-HOXA9 Decrease		Meth-HOXA9 Stable	Meth-HOXA9 Becomes Undetectable		Meth-HOXA9 Remains Undetectable	*p*-Value
From baseline to 2nd treatment cycle (*n* = 114)	17 (14.9%)	25 (21.9%)		38 (33.3%)	6 (5.3%)		28 (24.6%)	<0.001
OS; from treatment start	5.3 months		11.9 months			33.0 months		
From 2nd to 3rd treatment cycle (*n* = 99)	14 (14.1%)	4 (4.0%)		51 (51.5%)	2 (2.0%)		28 (28.3%)	<0.001
OS; from second treatment cycle	5.5 months		10.8 months			29.1 months		
From 3rd treatment cycle to evaluation (*n* = 89)	12 (13.5%)	6 (6.7%)		40 (44.9%)	5 (5.6%)		26 (29.2%)	0.050
OS; from third treatment cycle	5.5 months		12.1 months			24.7 months		

OS; overall survival.

## Data Availability

The data presented in this study are available on request from the corresponding author. The data are not publicly available due to privacy (General Data Protection Regulation, GDPR) and ethical restrictions.

## Supplementary Materials

The following supporting information can be downloaded at: https://www.mdpi.com/article/10.3390/cancers14071766/s1, Supplementary Materials; Table S1: Details on primers and probes; Table S2: Univariate Cox regression analyses; Table S3: Univariate Cox regression analyses in patients with high-grade serous carcinomas; Table S4: Multivariate Cox regression analyses in patients with high-grade serous carcinomas; Table S5: Correlation of meth-*HOXA9* dynamics after one cycle of treatment and meth-*HOXA9* status at baseline with Response Evaluation Criteria in Solid Tumours (RECIST) (A) and CA125 response (B). References [28,29] are cited in the Supplementary Materials.
