# Supplementary material for: Prognostic Impact of Circulating Methylated Homeobox A9 DNA in Patients Undergoing Treatment for Recurrent Ovarian Cancer
Source: Cancers (Basel). 2022 Mar 30;14(7):1766. doi: 10.3390/cancers14071766 (PMC8997085; doi:10.3390/cancers14071766)
Supplement: Supplementary file 1 [file cancers-14-01766-s001.zip › cancers-1643918-supplementary.pdf]

cancers

MDPI

Supplementary Materials

Prognostic Impact of Circulating Methylated Homeobox A9 DNA in Patients Undergoing Treatment for Recurrent Ovarian Cancer

Louise Faaborg [1,2,*], Rikke Fredslund Andersen [3], Marianne Waldstrøm [4], Jon Røikjær Henriksen [5], Parvin Adimi [1], Anders Jakobsen [1,2] and Karina Dahl Steffensen [1,2]

[1] Department of Oncology, Lillebaelt Hospital—University Hospital of Southern Denmark, Beriderbakken 4, 7100 Vejle, Denmark; parvin.adimi@rsyd.dk (P.A.); anders.jakobsen@rsyd.dk (A.J.); karina.dahl.steffensen@rsyd.dk (K.D.S.)

[2] Department of Regional Health Research, University of Southern Denmark, J.B. Winsløws Vej 19, 5000 Odense C, Denmark

[3] Department of Clinical Biochemistry and Immunology, Lillebaelt Hospital—University Hospital of Southern Denmark, Beriderbakken 4, 7100 Vejle, Denmark; rikke.fredslund.andersen@rsyd.dk

[4] Department of Pathology, Lillebaelt Hospital—University Hospital of Southern Denmark, Beriderbakken 4, 7100 Vejle, Denmark; marianne.waldstroem@rsyd.dk

[5] Department of Oncology, Odense University Hospital, J.B. Winsløws Vej 4, 5000 Odense C, Denmark; jon.henriksen@rsyd.dk

* Correspondence: louise.faaborg.larsen@rsyd.dk

Supplemental Materials

DNA Isolation and Quantification of DNA

Nine ml of blood was collected in EDTA tubes and plasma was isolated by centrifugation at 2000× *g* for 10 minutes within four hours after collection and stored at −80°C until analysis. Plasma was centrifuged again for 10 minutes at 10,000× *g* prior to purification. DNA was extracted from 4 mL plasma with the QiaSymphony purification system (Qiagen, Hilden, Germany) using the QIAsymphony DSP Circulating DNA kit (Qiagen, Hilden, Germany) as specified by the manufacturer. The purified ctDNA was eluted in 60 µl M-elution buffer after which 340 µl water was added to all samples. A qPCR for the *beta-2-microglobulin* gene (*B2M*) and Glycine max cysteine-rich Polycomb-like Protein (CPP1) was then performed to quantify the amount of DNA in each sample for quality control on a QuantStudio 12k flex system (Thermo Fisher Scientific, Waltham, MA, USA) [28]. Samples were concentrated to 20 µl on Amicon Ultra-0.5 Centrifugal Filter Units (Merck, Darmstadt, Germany).

HOXA9 Methylation Analysis

The purified DNA was bisulfite converted using the EZ DNA Methylation-Lightning Kit as recommended by the manufacturer (Zymo Research Corp., Irvine, CA, USA). Immediately after conversion the DNA was analyzed with an in-house designed *HOXA9* methylation specific assay and a control assay (Albumin) described in reference [29] using the BioRad® Droplet Digital PCR system QX200 (BioRad®, Hercules, CA, USA) according to the manufacturer's instructions.

Primers and *HOXA9* probes were purchased from LGC Biosearch technologies, Aarhus, Denmark and the albumin probe from Thermo Fisher Scientific (Waltham, MA, USA). Details on primer and probe sequences are listed in the table below (Table S1). Human methylated control DNA (Zymo Research Corp., Irvine, CA, USA), water and a pool of lymphocyte DNA from non-cancer individuals was included in each round of analyses as positive and negative controls.

PCR conditions:
Step 1 95 °C 10 min
Step 2 95 °C 15 sec 44 cycles
56 °C 1 min
Step 3 98 °C 10 min Sample ramp rate: 1.7 °C/sec

Table S1. Details on primers and probes.

Targeted Sequences	Fluoro-phore	Probe Sequence (5′>3′)	Conc. (μM)	Forward Primer Sequence (5′>3′) (4 μM)	Reverse Primer Sequence (5′>3′) (4 μM)
Albumin	VIC	AGGGTTTTTATAATTTA	0.4	GGGATGGAAAGAATTTTATGTT	AAACAAACTAACCCCAAATTCT
HOXA9	FAM	TTAGTTTAAGGCGACGGTGTT	0.2	GAGTATTTCGATTTTAGTTCGTGT	CGCGTACACTAAATTCCAC

After PCR amplification droplets were analyzed on a QX200 droplet reader (BioRad®, Hercules, CA, USA) and analyzed and quantified with QuantaSoft™ version 1.7.4 (BioRad®, Hercules, CA, USA). Meth-*HOXA9* ctDNA was reported as a percentage of total DNA ((meth-*HOXA9* copies/albumin copies) ×100) including a 95% confidence interval (CI) derived from a Poisson distribution. The limit of blank and cut-off for a positive sample was based on the analysis of blanks represented by two cohorts totaling 100 self- reported healthy volunteers. This resulted in a cut-off of ≥5 droplets containing meth-*HOXA9* equaling a positive test and samples with lower values were considered unmeasurable/undetectable. The same applied to samples with the 95% CI including 0.

Meth-*HOXA9* dynamics during treatment were considered stable if the 95% CI of the meth-*HOXA9* measurement was within the 95% CI of the previous measurement, decreasing if the measurement was below the 95% CI of the previous measurement and increasing if the 95% CI of the measurement was above the 95% CI of the previous measurement.

Table S2. Univariate Cox regression analyses. Factors entered in the multivariate Cox analysis had *p*-value <0.1 in the univariate Cox regression analysis.

	OS, Baseline (*n* = 126)		OS, Second Treatment Cycle (*n* = 114)		OS after Treatment Cycles (at Response Evaluation, *n* = 100)	
	**HR (95% CI)**	***p*-Value**	**HR (95% CI)**	***p*-Value**	**HR (95% CI)**	***p*-Value**
Meth-*HOXA9* status						
Undetectable	Reference		Reference		Reference	
Detectable	2.04 (1.29–3.23)	0.002	3.29 (1.95–5.55)	<0.001	2.54 (1.48–4.36)	0.001
Age (groups)						
<60 years	Reference		Reference		Reference	
60-70, 70-80 vs >80 years	0.96 (0.76–1.21)	0.722	1.02 (0.78–1.33)	0.907	0.956 (0.72–1.28)	0.755
FIGO stage at diagnosis						
I–II	Reference		Reference		Reference	
III–IV	2.41 (0.76–7.65)	0.134	3.08 (0.75–12.60)	0.117	2.81 (0.68–11.53)	0.152
Histology						
Serous	Reference		Reference		Reference	
Non-serous	0.79 (0.42–1.49)	0.473	0.71 (0.34–1.49)	0.364	0.47 (0.19–1.17)	0.105
BMI						
>25	Reference		Reference		Reference	
<25	0.85 (0.56–1.30)	0.458	0.75 (0.47–1.20)	0.229	0.75 (0.45–1.25)	0.277
BRCA status						
BRCA1/2	Reference		Reference		Reference	
Unknown *BRCA* status + *BRCA* wild type	0.60 (0.32–1.13)	0.113	0.59 (0.29–1.18)	0.136	0.62 (0.29–1.30)	0.206
Performance status						
0–1	Reference		Reference		Reference	
2	2.99 (1.89–4.73)	<0.001	2.44 (1.39–4.28)	0.002	2.48 (1.31–4.71)	0.005
Platinum sensitive						
No	Reference		Reference		Reference	

Yes	0.38 (0.24–0.61)	<0.001	0.43 (0.26–0.71)	0.001	0.50 (0.29–0.85)	0.011
Previous lines of chemotherapy						
1–3	Reference		Reference		Reference	
4–5	5.07 (2.70-9.51)	<0.001	6.23 (3.03-12.80)	<0.001	7.21 (3.23-16.10)	<0.001
CA125 (kUI/L), at baseline						
>500 kUI/L	Reference		Reference		Reference	
≤500 kUI/L	0.62 (0.41–0.93)	0.021	0.61 (0.39–0.97)	0.037	0.63 (0.38–1.03)	0.064

OS, Overall survival; HR, Hazard ratio; CI, Confidence interval.

Table S3. Univariate Cox regression analyses in patients with high-grade serous carcinomas.

	**OS, Baseline (*n* = 108)**		**OS, Second Treatment Cycle (*n* = 98)**		**OS after Treatment Cycles (at Response Evaluation, *n* = 88)**	
	**HR (95% CI)**	***p*-Value**	**HR (95% CI)**	***p*-Value**	**HR (95% CI)**	***p*-Value**
Meth-*HOXA9* status						
Undetectable	Reference		Reference		Reference	
Detectable	1.60 (1.00–2.58)	0.052	2.73 (1.58–4.73)	<0.001	2.43 (1.38–4.30)	0.002
Age (groups)						
<60 years	Reference		Reference		Reference	
60–70, 70–80 vs >80 years	0.97 (0.76–1.24)	0.809	1.05 (0.80–1.39)	0.710	1.00 (0.74–1.34)	0.982
FIGO stage at diagnosis						
I–II	Reference		Reference		Reference	
III–IV	3.84 (0.94–15.63)	0.062	3.30 (0.81–13.56)	0.097	3.10 (0.75–12.78)	0.117
BMI						
>25	Reference		Reference		Reference	
<25	0.77 (0.48–1.23)	0.275	0.71 (0.42–1.19)	0.193	0.70 (0.40–1.21)	0.198
BRCA status						
BRCA1/2	Reference		Reference		Reference	
Unknown *BRCA* status + *BRCA* wild type	0.57 (0.30–1.08)	0.087	0.56 (0.27–1.13)	0.104	0.56 (0.26–1.18)	0.129
Performance status						
0–1	Reference		Reference		Reference	
2	3.31 (2.01–5.46)	<0.001	2.81 (1.56–5.07)	0.001	2.75 (1.40–5.38)	0.003
Platinum sensitive						
No	Reference		Reference		Reference	
Yes	0.38 (0.22–0.64)	<0.001	0.43 (0.25–0.75)	0.003	0.47 (0.26–0.85)	0.012
Previous lines of chemotherapy						
1–3	Reference		Reference		Reference	
4–5	5.21 (2.69–10.09)	<0.001	7.04 (3.38–14.70)	<0.001	7.08 (3.13–15.97)	<0.001
CA125 (kUI/L), at baseline						
>500 kUI/L	Reference		Reference		Reference	
≤500 kUI/L	0.57 (0.37–0.89)	0.013	0.56 (0.34–0.91)	0.018	0.58 (0.35–0.98)	0.040

OS, Overall survival; HR, Hazard ratio; CI, Confidence interval.

Table S4. Multivariate Cox regression analyses in patients with high-grade serous carcinomas. Factors entered in the multivariate Cox analysis had *p*-value <0.1 in the univariate Cox regression analysis.

	**OS, Baseline (*n* = 108)**		**OS, Second Treatment Cycle (*n* = 98)**		**OS after Treatment Cycles (at Response Evaluation, *n* = 88)**	
	**HR (95% CI)**	***p*-Value**	**HR (95% CI)**	***p*-Value**	**HR (95% CI)**	***p*-Value**
Meth-*HOXA9* status						
Undetectable	Reference		Reference		Reference	
Detectable	1.64 (1.00–2.68)	0.050	2.53 (1.43–4.50)	0.002	2.00 (1.06–3.77)	0.033
Performance status						
0–1	Reference		Reference		Reference	
2	3.47 (2.02–5.96)	<0.001	2.90 (1.55–5.44)	0.001	2.86 (1.38–5.96)	0.005
Platinum sensitive						
No	Reference		Reference		Reference	
Yes	0.48 (0.27–0.84)	0.010	0.49 (0.27–0.90)	0.021	0.77 (0.40–1.49)	0.437
Previous lines of chemotherapy						
1–3	Reference		Reference		Reference	
4–5	2.87 (1.45–5.68)	0.002	3.83 (1.79–8.23)	0.001	3.53 (1.46–8.53)	0.005
CA125 (kUI/L), at baseline						
>500 kUI/L	Reference		Reference		Reference	
≤500 kUI/L	0.68 (0.42–1.09)	0.108	0.81 (0.47–1.38)	0.433	0.70 (0.40–1.24)	0.222

OS, Overall survival; HR, Hazard ratio; CI, Confidence interval.

Table S5. Correlation of meth-*HOXA9* dynamics after one cycle of treatment and meth-*HOXA9* status at baseline with Response Evaluation Criteria in Solid Tumours (RECIST) (A) and CA125 response (B).

**Response Evaluation by Imaging (RECIST)**	**Partial Response**	**Stable Disease**	**Progression**	***p*-Value**
	A			
Imaging after three cycles and meth-*HOXA9* at baseline (*n* = 97)				
Meth-*HOXA9* detectable (*n* = 63)	23 (36.5%)	25 (39.7%)	15 (23.8%)	
Meth-*HOXA9* undetectable (*n* = 34)	14 (41.2%)	14 (41.2%)	6 (17.6%)	0.769
Imaging after three cycles and meth-*HOXA9* after one cycle (*n* = 96*)				
Meth-*HOXA9* increase (*n* = 11)	1 (9.1%)	1 (9.1%)	9 (81.8%)	
Meth-*HOXA9* stable or decrease (*n* = 54)	23 (42.6%)	21 (38.9%)	10 (18.5%)	
Meth-*HOXA9* undetectable (*n* = 31)	12 (38.7%)	17 (54.8%)	2 (6.5%)	<0.001
**Response evaluation by CA125 (GCIG)**	**CA125 response**	**Stable CA125**	**CA125 progression**	***p*-value**
	B			
Evaluable CA125 and meth-*HOXA9* at baseline (*n* = 83)				
Meth-*HOXA9* detectable (*n* = 60)	25 (41.7%)	18 (30.0%)	17 (28.3%)	
Meth-*HOXA9* undetectable (*n* = 23)	9 (39.1%)	7 (30.4%)	7 (30.4%)	0.974
Evaluable CA125 and meth-*HOXA9* after one cycle (*n* = 81**)				
Meth-*HOXA9* increase (*n* = 13)	1 (7.7%)	4 (30.8%)	8 (61.5%)	
Meth-*HOXA9* stable or decrease (*n* = 49)	22 (44.9%)	16 (32.7%)	11 (22.4%)	
Meth-*HOXA9* undetectable (*n* = 19)	11 (57.9%)	3 (15.8%)	5 (26.3%)	0.017

*One and **two patients had missing meth-*HOXA9* value at treatment cycle two.

References

28. Pallisgaard, N.; Spindler, K.L.G.; Andersen, R.F.; Brandslund, I.; Jakobsen, A. Controls to validate plasma samples for cell free DNA quantification. *Clin. Chim. Acta* **2015**, *446*, 141–146.
29. Roperch, J.P.; Incitti, R.; Forbin, S.; Bard, F.; Mansour, H.; Mesli, F.; Baumgaertner, I.; Brunetti, F.; Sobhani, I. Aberrant methylation of NPY, PENK, and WIF1 as a promising marker for blood-based diagnosis of colorectal cancer. *BMC Cancer* **2013**, *13*, 1–10, doi:10.1186/1471-2407-13-566.
